# Foreign Medico-Psychological Literature

**Published:** 1862-07

**Authors:** 


### FOREIGN MEDICO-PSYCHOLOGICAL LITERATURE.

Our Retrospect of current Foreign Medico-Psychological Literature embraces the following subject:

*Upon some forms of Insanity which could not with justice be committed to Hospitals for the Insane.* By Dr. Salomon; Samosczyn.

*Aërophobia.*—There is not only hydrophobia, but an aërophobia. Without here setting both phenomena parallel, it is enough to characterize the latter, and to pursue the subject, when it, leaving material grounds, passes into insanity, and outgrows trivial discipline and treatment. Of course, we must first put aside the many irritations of the skin as they occur in fever, in catarrh, in asthma, in rheumatism, and other diseases; as in these cases the shrinking from the atmospheric air is merely a subordinate symptom of the disease affecting the whole body, or a special organ; at least it has always hitherto been considered so. It is also difficult in a detailed account to enter into all the irritations of the skin which are exhibited in hypochondria, hysteria, melancholia, and in the first stage of mania, and especially so because the physiological side of this group of appearances has not been sufficiently discussed to enable it to be ascertained in how far in one particular case the abnormal cold sensation is the cause or symptom of derangement. The psychologist at least, has, to my knowledge, always looked upon every cold sensation as a purely eccentric symptom, although different considerations might lead to the conclusion that, oftener at least than is commonly supposed, derangement of the *steinsasthesia* may be considered in the light of a cause. If we consider, in the first place, the extensive diffusion of the nerves of the skin organs, in order that we may estimate in a rough way only their probably material importance in relation to the remaining nervous system, we may easily imagine that a derangement in the sensation of the skin might also bring about a derangement in the central organs of the nervous system. If we pass with the philosopher Noak to a deeper and more exact appreciation of the *vehicles* of common sensation as an indispensable means of help, nay, even as an integral component part of the intelligent portion of the brain, we are led involuntarily to think of the pathological theory according to which the burning of the third part of the surface of the skin produces death; and how seldom is it taken into consideration, how great an influence a derangement of the sensibility of a much greater surface of the skin may exercise upon the power of perception of the brain. If, indeed, insanity be once there, the abnormal state of the skin will scarcely be considered worthy of attention in comparison with the anormal phenomena of the brain; although the care which is bestowed upon the skin of the insane in asylums proves that this item is not left out of account in practice. Moreover, the old theory of a causative relation of pellagra and insanity would probably proceed from a dim idea of the importance of a healthy state of the skin. Sickness would be more interesting, if in cases of disease the direct connexion of central *irritation* with the *irritation* of the sensibility of the skin was proved. There are, however, so many cases in which the one irritation occurs and passes away without any perceptible derangement of the mind, that an opportunity for observation is seldom afforded to the psychologist. Such patients, however, give us physicians in private practice much trouble, and cause us to wish often enough for the material apparatus and approved means of carrying out treatment psychologically. The next division of patients corresponds to the title at the top of these lines, which saves us from giving a somewhat more exact explanation of what we think "aërophobia." First of all, we must guard ourselves from in any way conveying the idea that we have ever observed a case in which idiopathic irritation of the nervous system was transmitted to the brain without a causative connexion with some other condition of disease. The peculiarity in the observed cases was, that every unhealthy condition had already ceased months and years previous, and that the aërophobia remained as a single isolated symptom of the earlier disease. Such patients guarded themselves with the greatest care against keen air, against draughts, against wind, went seldom out, and only in the warmest weather, and covered by many clothes; they lay for years under large beds, and considered those their bitterest foes who advised them to abandon their place of rest. Many perspire much, and thereby become still more sensitive. The window is not allowed to be opened, the door only with the greatest caution. With this nervous dread of contact with the cold air is associated sooner or later, in many cases, a transmission of the irritation of the skin to the mucous membrane, to the nervous expansion of the nerves of smell in the mucous membrane of the nose, producing hyperosmia; or passing to the nerves of the mucous membrane of the chest, produces difficulty of breathing—phenomena which are reproduced by cold, or any unpleasant unusual smell. If neither cold nor unpleasant smells recal these agents to the remembrance of the patient, he remains cheerful and feels himself well. Other diseased conditions are not to be discovered. In two of my patients it was preceded for months and years by feverish catarrh; another suffered for years from an affection of the liver, and complained often of cold feet and prolonged shivering. Asthma is a very common concomitant of this disease, and I once attended a patient who would not allow a fire to be lit during the whole winter, preferring to be encircled by a perfect wall of beds, coverlets, &c., because he imagined himself unable to bear the smell of the fuel in a well-ventilating stove. This condition continues, if it does not receive a check, till the end of life; the patient becomes ever more susceptible to fresh air, and more anxious to protect himself from the cold; medical aid becomes unavailable, and if death does not ensue, it may easily be imagined that from such an unnatural life the saddest consequences result. It is evident from the foregoing description that the laws of nervous physiology hold good in such affections; that in many important diseases these affections of the nerves may continue long after the original cause of suffering has disappeared (tetanus epilepsia). Aërophobia is connected with catarrh, that hyperæsthesia of the skin which induces the patient alternately to approach and recede from the stove. The catarrh disappears, the dysæsthesia of the skin may remain. In the same way it is connected with disease of the lower parts of the body, which is often accompanied by an irritation of the skin. The following cases will serve for the elucidation of the foregoing:—

I. On the 29th April, 1859, I was called to see a shepherdess in the neighbourhood, who had been confined to bed for eighteen weeks, and whose case had neither been cured nor ameliorated by the use of various medicines. I found a woman about 51 years of age; a very fat, and in spite of her long confinement to bed, strong-looking person, the mother of several children, and one who had previously enjoyed good health. She lay in bed enveloped in numerous petticoats, dresses, and jackets, and covered up to her nose by one of those heavy upper beds which are used in that part of the country. Her own account was, that eighteen weeks before she had been seized by a feverish cold, which obliged her to keep her bed. Since that time she had felt very weak and "unable to bear the air," because, as she expressed it, it made her shiver violently all over. Thus she suffered much from recurring attacks of pain in the limbs, and complained of shooting pains through the whole body, perspired much (she had often taken medicine on this account), shivered before my eyes when she was uncovered, that I might examine her, and was obliged to be soon re-covered. The examination, after all accuracy, showed not the slightest disease. Besides, she had a very good appetite, sufficient to supply the requisite nourishment, and had a normal temperature of the skin. The courses had long ago ceased. The sleep was good, and undisturbed by pain. The object now was to allay that excessive sensibility of the skin. This, however, could not be attained through the ordinary means, because the patient, at the simple order to leave her bed, fancied she had not the power to do so; in fact, considered that the mere lifting of the bed-clothes would infallibly produce death. The next object was, therefore, to remove this prejudice. I am ashamed to confess that I called to aid omnipotent humbug, and certainly with the most decided results. I asked the woman if she knew when the new moon would be? She did not know, and a calendar had to be brought, from which we learned that the new moon would appear on the night of the 1st of May, three days from that time. So I ordered her on the last of these days, at six o'clock in the morning, punctual to the moment by a well-regulated clock, to allow herself to be carried outside the door, and placed upon a bench. To prevent cold, she was to be covered with warm skins. When she had been there exactly one half hour by the clock she was to be carried back to bed. This was to be repeated on the two following days. On the next day she was to sit for an hour, and walk exactly 100 steps; on the following one 200 steps; and so on until 1000 steps were achieved. After that she would be perfectly well, if every three days she took off a petticoat or jacket, and caused it to be hung on the west side of her apartment. As I have already intimated, the punctual attention to my orders resulted in a complete cure, and the woman has been perfectly well ever since.

An almost identical case was treated by me in my former place of residence, Schneidermühl. This case amongst others may prove that in many points it should be dealt with as a purely psychological disease. If we were sufficiently enlightened and supplied with good asylums, to which such patients might he sent without encountering violent opposition, the necessity for resorting to such humbug would be avoided, and the case more suitably treated under the superintendence of the chief physician. Not merely maniacs and melancholics, but also the so-named hypochondriacs, would be more easily treated and cured by isolation.

II. S. S., 72 years of age, has been already treated by me seven years, and by my predecessor thirteen years, and was ill fifteen years before that. He is in good circumstances, a clever honest man, who had had a hard struggle in his youth. Easier circumstances were accompanied by sickness. Thirty years ago he was attacked, most probably as the result of a cold, by liver complaint, accompanied by an asthmatical affection. After a variety of treatment, the complaint was subdued at Carlsbad—that is, the liver complaint, not the asthma. From his description of the latter, it is probable that the swollen liver had pressed partly upon the diaphragm and then upon the nervus phrenicus, and partly upon the nervous plexus of the stomach, causing the difficulty of breathing, which he and his physicians called asthma. Whether the true pathognomical conditions of asthma were ever there, is very questionable. Neither I nor my predecessor, Dr. Lowenthal, Posen, had ever, except once during a period of twenty years, observed any other symptom of oppression than a mere cramp, which extended from the scrobiculus cordis along the vagus as far as the manubrium sterni, but seldom higher. This continued for days and weeks without variation; was accompanied by the discharge of pure urine, and only seldom terminated in a cough. When there was any question of business, news, or relations of any interest, we heard nothing of the oppression, and even during the attacks the voice was sonorous and powerful. Digestion was healthy, and attention was only called to it because the patient voluntarily imposed upon himself his old diet of Carlsbad, coffee and soup, which produced a coating on the tongue; it became clean again on his return, after the attack, to ordinary and solid food. Stools perfectly normal, and the use of aperients produced a sensation of weakness bordering upon faintness. The feeling of oppression was felt as soon as the patient fancied he had inhaled a breath of cold air, whether he had been exposed to it or not; or as soon as he perceived an unpleasant odour he went immediately to bed and put on a fly blister, from which he imagined he experienced relief. The surface of his breast had the appearance of being flayed, and as often as I persuaded him not to apply the blister because it produced dysury, the attack was sure to continue, until, with or without my leave, the blister was applied. Then would succeed several days, or even weeks, of relief, until he met with some annoyance, encountered a current of cold air, smelt a cigar, or passed a newly scoured room. Now, touching the *hyperosmia*, I once went intentionally to him when the overcoat which I had on had been accidentally smeared with tar. I went straight to him with an exciting piece of news, and continued with him in animated conversation for two hours without his once perceiving this very penetrating smell. He only asked me incidentally if I had been giving musk to any patient, for there was an odour like it. When I told him, several days afterwards, that I had had tar on my coat, which had smelt so very strongly without giving him an illness he was quite offended, and did not call me in for a long time. He is generally very well in the heat of summer, walks actively in the streets, drives out occasionally, has good spirits, enjoys his food, takes great interest in his business; but as soon as winter draws near, he spends the most of his time in bed, keeps a strict guard over the keeping up of the fire, in case it should become too cold, will not allow the windows or even the door of a press in his room to be opened, and if he ever does venture into his sitting or business room, it is only in the mildest weather, when he is sure that the house doors are all shut, and with a handkerchief before his mouth. In this way he has passed more than thirty years. Five years ago I sent him to Colberg, in order to breathe the sea air and to get his skin hardened; after ten days he came back, because "the air was too sharp for him." Accidentally several very mild winters followed, and yet had the advantage that during them "business" was very active and prosperous. Last winter, on the contrary, was again very bad, and the aërophobia reassumed the most extraordinary caprices. Towards spring it was accompanied by an acute bronchial catarrh, which ran the usual course, and which, in spite of the oppression and cramp of which he complained, showed no symptoms of asthma. So far as his physical condition is concerned, neither I nor any other physician have ever seen, with the exception of the above-mentioned catarrh, any disease in the lower part of the body, in the lungs, or in the heart of this robust old man.

At present the patient has been for five weeks at Colberg, where, according to the last news, he has been entirely free from oppression, and has been making excursions on the sea, even in pretty rough weather. "That is just a common case of hypochondria," I hear many remark. That is certainly the usual term applied; but names do not always express ideas. It is sufficient for me to have proved, by the history of this case, that after a disease of the liver, &c., had run its course, a solitary symptom of the same abnormal feeling of cold remained for thirty years, and by reaction upon the brain has produced abnormal sensations in it, so that the patient, not in a trivial sense, but virtually, must be numbered amongst the insane.—*Allgemeine Zeitschrift für Psychiatrie und Psychisch. gerichtlecht Medecin.*

